# Translation of the 27-gene immuno-oncology test (IO score) to predict outcomes in immune checkpoint inhibitor treated metastatic urothelial cancer patients

**DOI:** 10.1186/s12967-022-03563-9

**Published:** 2022-08-16

**Authors:** Robert S. Seitz, Michael E. Hurwitz, Tyler J. Nielsen, Daniel B. Bailey, Matthew G. Varga, Brian Z. Ring, Carrie F. Metts, Brock L. Schweitzer, Kimberly McGregor, Douglas T. Ross

**Affiliations:** 1 Oncocyte, Inc., Irvine, California USA; 2 Yale Cancer Center/Smilow Cancer Hospital, New Haven, Connecticut USA

**Keywords:** Immuno-oncology, Immunotherapy, Biomarker, Tumor immune microenvironment, Bladder cancer, Metastatic urothelial cancer, Immune checkpoint inhibitors

## Abstract

**Background:**

The IO Score is a 27-gene immuno-oncology (IO) classifier that has previously predicted benefit to immune checkpoint inhibitor (ICI) therapy in triple negative breast cancer (TNBC) and non-small cell lung cancer (NSCLC). It generates both a continuous score and a binary result using a defined threshold that is conserved between breast and lung. Herein, we aimed to evaluate the IO Score’s binary threshold in ICI-naïve TCGA bladder cancer patients (TCGA-BLCA) and assess its clinical utility in metastatic urothelial cancer (mUC) using the IMvigor210 clinical trial treated with the ICI, atezolizumab.

**Methods:**

We identified a list of tumor immune microenvironment (TIME) related genes expressed across the TCGA breast, lung squamous and lung adenocarcinoma cohorts (TCGA-BRCA, TCGA-LUSQ, and TCGA-LUAD, 939 genes total) and then examined the expression of these 939 genes in TCGA-BLCA, to identify patients as having high inflammatory gene expression. Using this as a test of classification, we assessed the previously established threshold of IO Score. We then evaluated the IO Score with this threshold in the IMvigor210 cohort for its association with overall survival (OS).

**Results:**

In TCGA-BLCA, IO Score positive patients had a strong concordance with high inflammatory gene expression (p < 0.0001). Given this concordance, we applied the IO Score to the ICI treated IMvigor210 patients. IO Score positive patients (40%) had a significant Cox proportional hazard ratio (HR) of 0.59 (95% CI 0.45–0.78 p < 0.001) for OS and improved median OS (15.6 versus 7.5 months) compared to IO Score negative patients. The IO Score remained significant in bivariate models combined with all other clinical factors and biomarkers, including PD-L1 protein expression and tumor mutational burden.

**Conclusion:**

The IMvigor210 results demonstrate the potential for the IO Score as a clinically useful biomarker in mUC. As this is the third tumor type assessed using the same algorithm and threshold, the IO Score may be a promising candidate as a tissue agnostic marker of ICI clinical benefit. The concordance between IO Score and inflammatory gene expression suggests that the classifier is capturing common features of the TIME across cancer types.

**Supplementary Information:**

The online version contains supplementary material available at 10.1186/s12967-022-03563-9.

## Background

Immune checkpoint inhibitors targeting programmed cell death 1 or its ligand, programmed cell death 1 ligand 1, (PD-1 or PD-L1, respectively) are active in many different tumor types and may modulate the immune response by impacting a common immune regulatory pathway. To date, observed benefit across tumor types has been modest despite co-development of candidate biomarkers for identifying responders to ICI therapy, including immunohistochemistry (IHC) for PD-L1 and tumor mutation burden (TMB). These biomarkers are limited in their value as predictive markers due to inconsistent scoring methods and differing thresholds for positivity, often specifically tailored to tumor types or even clinical studies [1–3]. Additionally, ICIs are a systemic therapy which are impacted by the TIME [4–6]. Therefore, a biomarker that provides a phenotypic classification of the TIME may be useful to better identify tumors poised to respond to ICI therapeutics.

Atezolizumab was the first FDA approved ICI in mUC based on the initial results of IMvigor210, a non-randomized Phase II clinical trial involving two different cohorts of mUC. Cohort I (NCT02951767) was comprised of 119 patients who were ineligible to receive cisplatin [7] and Cohort II (NCT02108652) was comprised of 310 patients whose disease had progressed on or after platinum therapy [8]. Based on this study, atezolizumab was approved in May of 2016 under an accelerated approval pathway for platinum refractory bladder cancers (Cohort II) independent of PD-L1 status even though the overall response rates were more favorable in those patients who had PD-L1 staining in  ≥ 5% in immune cells (IC2/3). Unfortunately, a Phase 3 confirmatory study, IMvigor211 (NCT02302807), using the primary endpoint of OS where patients with IC2/3 status were tested first in a hierarchical sequence, failed to reach significance [9]. In 2018, Mariathasan and colleagues published the gene expression data for 348 of IMvigor210 patients, representing a mix of both cohorts, along with exploratory analyses for the association with outcome using candidate genomic biomarker signatures and clinical features in a search for biomarkers that might inform response [10].

The IO Score has been validated as a biomarker that identifies patients likely to benefit from ICIs in TNBC and NSCLC [11–15]. These studies were conducted across tumor types using the same threshold of positivity for IO Score. The IO Score was derived from an unsupervised classification of triple negative breast cancer (TNBCtype) specimens [14, 16, 18]. Two subtypes originally thought to describe intrinsic features of the tumor (the immunomodulatory or IM and the mesenchymal stem-like or MSL, respectively) were later recognized as classifiers that distinguish tumor infiltrating lymphocytes and stromal features of a particular tumor’s TIME, respectively. A third subtype, the mesenchymal (M), likely reflects tumors that have undergone some level of epithelial-to-mesenchymal transition (EMT) [17, 19], which in turn is associated with tumor resistance to the immune system [20–24]. Together, the unique interaction of these components defines the IO Score.

While the IO Score was developed in TNBC, its three components are not unique to TNBC but collectively measure ubiquitous features across tumors of epithelial origin. Given that urothelial carcinoma is likewise of epithelial origin, we sought to test the clinical utility of the IO Score to identify patients who benefit from the administration of ICIs in bladder cancer. We first evaluated the predefined IO Score threshold using the IM, MSL, and M subtypes to classify ICI naïve samples from TCGA-BLCA. Using the predefined threshold for IO Score we then tested its association with treatment outcome in patients from the IMvigor210 study. Finally, we compared these results to the previously published extensive exploratory analysis of candidate biomarkers, biomarker signatures, and clinical factors [10].

## Methods

### Data access, software and statistical calculations

R version 4.0.2 (2020-06-22) and R-Studio Version 1.0.143 were used for all data manipulation and calculations [25, 26]. Heatmaps were generated using the ComplexHeatmap package in R [27]. TCGA datasets containing the whole transcriptome RNA-seq gene expression data for TCGA-BRCA, TCGA-LUAD, TCGA-LUSC, and TCGA-BLCA were downloaded using the GenomicDataCommons and TCGAbiolinks packages in R [28–31]. The expression data was filtered for identifiable HUGO Gene Nomenclature Committee (HGNC) gene symbols, non-redundancy, and variation across tumor samples. If a single gene had multiple transcripts which met the above criteria the expression across samples were averaged and this, depending upon the dataset, resulted in approximately 34,000 uniquely differentiated members. The four data sets were scaled and log transformed.

The IMvigor210 data including gene expression, clinical data, and alternative biomarker signatures, were made available by the IMvigor210 investigators [10] who created a customized analysis package in R IMvigor210 CoreBiologies [32], which was utilized to download the entire dataset and normalize the gene expression. The R packages survival [33], prodlim [34], and MASS [35] were used for all survival analyses, median follow-up and ordinal regression, respectively. Chi-square testing was used to determine significance testing when comparing response groups and other categorical variables. Bivariate testing comparing the IO Score with clinical factors or genomic signatures was performed by adding both factors as independent variables to a Cox proportional hazard equation. When performing bivariate analysis for comparison between IO Score and the genomic signatures explored by [10], and a binary threshold was not defined for the latter, the median value was used.

Kaplan–Meier estimates were used to calculate median OS and percentage alive at end of study (24.5 months), and Cox proportional hazards was used to calculate hazards with the Efron method used for handling ties.

### Assessment of the established IO score threshold in bladder cancer TIME classification

**Fig. 1:**
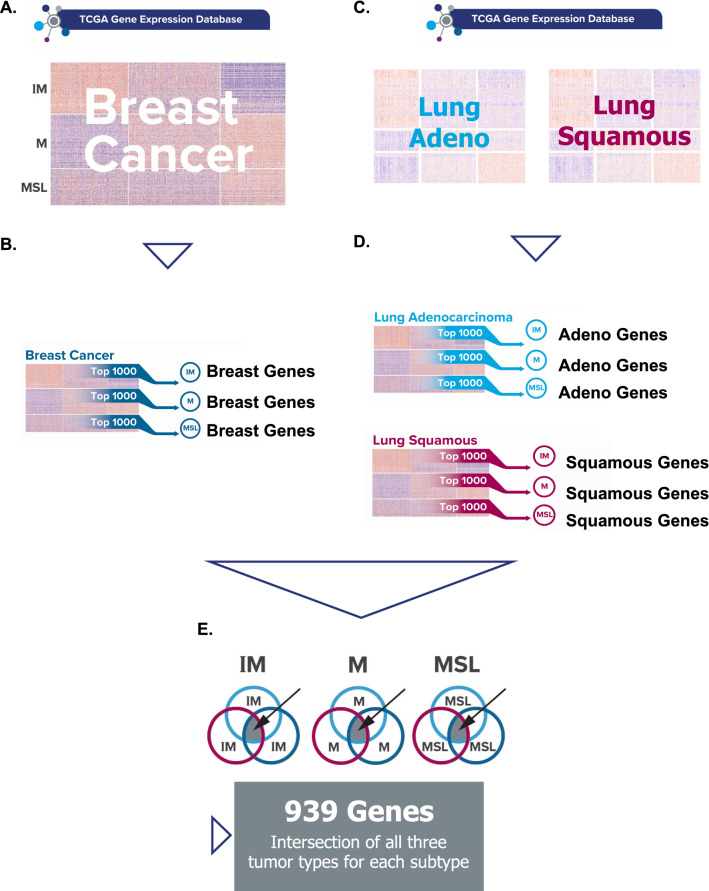
Isolating a Tumor Immune Microenvironment (TIME) Gene Set **A** Downloaded the full dataset of gene expression data in Breast Cancer from the TCGA Database; **B** Compared against an established 101-gene molecular classifier for TNBC [18] to correspond with three known signatures: IM, M, and MSL, and selected the top 1,000 genes correlated with each of the IM, M, and MSL signatures; **C** Repeated with TCGA gene expression data sets from Lung Adenocarcinoma and Lung Squamous Cell Carcinoma; **D** Identified top 1,000 genes for each signature in each additional cancer dataset; **E** Intersection of top 1,000 genes in each subtype between three cancers yielded a total of 939 genes

Expression data from TCGA—TCGA-BRCA, TCGA-LUSQ, and TCGA-LUAD—were log transformed and then scaled by patient and gene axes. The 101-gene centroids for the IM, M, and MSL subtypes were used to generate patient specific scores in TCGA-BRCA, TCGA-LUAD, TCGA-LUSQ [18] for each classification. The expression pattern of each of the 34,000 protein coding and non-coding transcripts across each tissue type dataset were then compared to the IM, MSL and M patient phenotypes (Pearson correlation coefficient). The top 1000 correlated genes for each subtype in each of the three tissues were selected. All selected genes were statistically significant but no correction for multiple hypotheses was performed. The three gene sets were compared and the 939 that were common between all three selected gene sets was selected as the expanded TIME classifier gene set (Fig. 1).

**Fig. 2:**
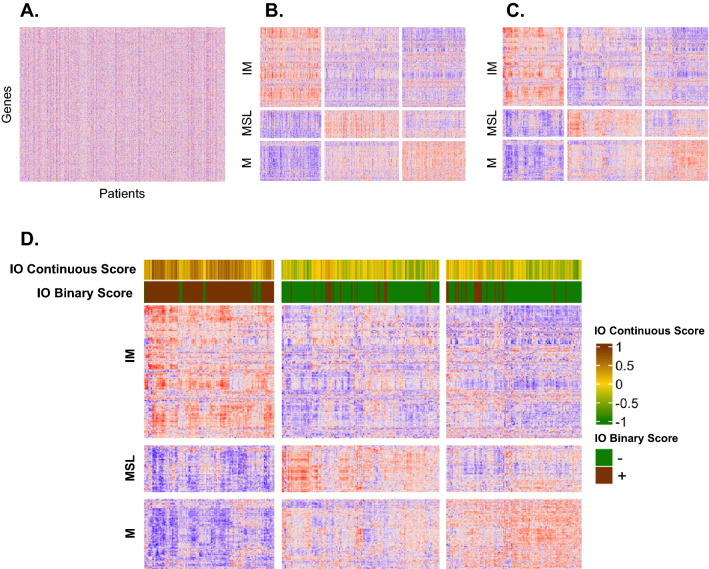
Process of Clustering TCGA Bladder Cancer Data by TIME Phenotype **A** Unclustered TCGA bladder cancer RNA-Seq data set of the previously identified 939 TIME genes; **B** Patients are put into three groups on both the x- and y-axes via k-means clustering; **C** Hierarchical clustering is performed within each of the three groups on the x- and y-axes, respectively; **D** Each patient’s IO Score is overlaid above the heatmap, both as a continuous variable (top bar) and as a binary score with a threshold of 0.09

The TCGA-BLCA bladder cancer gene expression was filtered to these 939 genes (Fig. 2A). Each of the 406 bladder cancer tumor samples were assigned a phenotypic score for each of IM, MSL, and M using the 101 gene centroids. K-means clustering was performed for the 939 genes with k = 3, using supplied centers created with these tumor phenotypic scores. This assigned each of the 939 genes to one of the IM, M, or the MSL clusters. K-means was performed on the x-axis (tumor samples), with k = 3 and the centers derived by traditional k-means methodology (Fig. 2B). Hierarchical clustering was performed within the IM, M and MSL k-means gene clusters on the y-axis and the three k-means tumor clusters on the x-axis. (Fig. 2C).

The gene expression data for the 27 genes used to generate the IO Score were extracted and the IO algorithm was applied to the 406 TCGA-BLCA tumor samples, giving each sample a continuous score (range of − 1 to 1) then, a binary score (IO Score + /IO Score-) if this continuous score was equal or greater than the previously established threshold of 0.09 (Fig. 2D). Thresholds were evaluated by determining the value where the difference between sensitivity and specificity was minimized [36]. Mathematically, this can be expressed as the minimum value of the output of the following equation:

$$\sum_{1}^{Total \,Samples}absolute\, value(sensitivity\left(x\right) - specificity(x))$$

where x represents applying every possible threshold to the 406 IO Scores from the TCGA-BLCA tumor samples. We then calculated the accuracy with both the existing threshold and the calculated threshold and tested their difference by a statistical test for proportions.

### Analysis of the IMvigor210 data

The whole transcriptome gene expression, and clinical data, including response and survival data, from the IMvigor210 study have been released for 76 of the platinum contra-indicated Cohort I patients and 272 of the platinum refractory Cohort II patients for a total of 348 patients [10]. The 27 gene IO Score was calculated as previously described [14] using the pre-established threshold of positivity to classify patients as IO Score positive or negative. Kaplan–Meier curves were plotted to estimate OS for IO Score and Cox proportional hazard analysis was used to calculate the HR and 95% CIs. The mean of the continuous variable of the IO Score was compared to four categories of objective response—complete response, partial response, stable disease, and progressive disease (CR, PR, SD, and PD, respectively) using either a t-test of means between groups or ordinal regression for a trend in all four groups. Finally, the IO Score was tested in a series of bivariate models with reported clinical factor and relevant biomarker classification and the Cox proportional hazard method was used to calculate HRs using the log-rank method for significance.

## Results

**Fig. 3:**
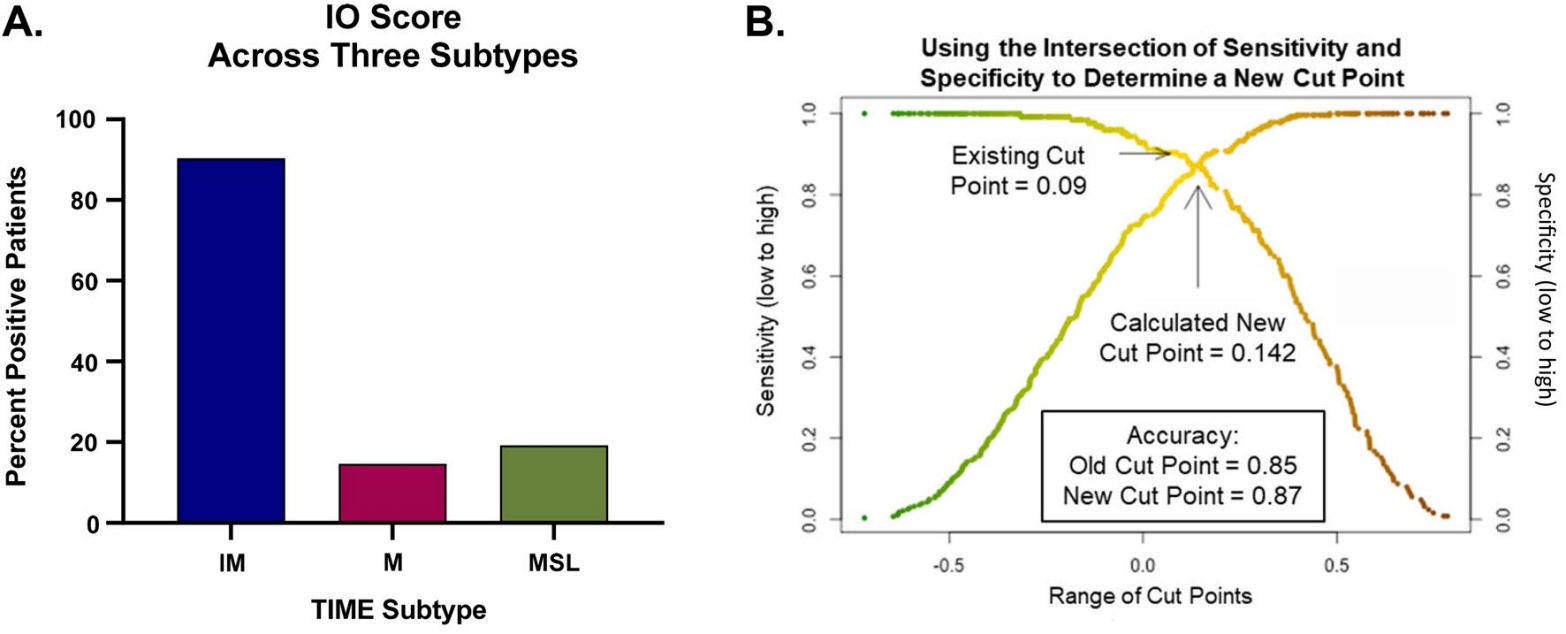
IO Score Positivity Among TIME Phenotype and Confirming Binary IO Score Threshold **A** Percentage of IO Score positive cases in each TIME phenotype (IM, MSL, and M); **B** Calculation of a new theoretical threshold for positivity of the IO Score. Positive IO Score in the IM phenotype and negative scores in the M and MSL phenotypes are considered true positives and true negatives, respectively. Using the intersection of sensitivity and specificity where both the actual threshold and the new theoretical threshold are then used to calculate overall accuracy

The TCGA-BLCA tumor samples were classified into IM, MSL, and M subtypes using the expanded TIME classifier. We then compared these classifications against the IO continuous and binary scores using the IM subtype (high inflammatory gene expression) as the surrogate classifier for IO Score positive (Fig. 2D). In total, 125 of 406 TCGA-BLCA tumor samples clustered with the high IM expressing genes. The MSL and M subtypes were surrogates for IO Score negative. In total, 151 patients clustered with the MSL genes, and 130 patients clustered with the M genes. The distribution of the IO Score positive patients within each of these clusters was 113 (90.4%), 22 (14.6%), and 25 (19.2%) for IM, MSL, and M respectively, showing a strong concordance between IO Score positive patients and the IM subtype (Fig. 3A, p < 0.0001, chi-squared test).

In order to confirm the predefined IO Score threshold we generated a threshold specific to the TCGA-BLCA cohort by comparing the minimal distance between sensitivity and specificity for classification of IO Score positive patients (IM) versus IO Score negative patients (M or MSL) and obtained a theoretical, optimal binary classification threshold of 0.142 (Fig. 3B) [36]. The overall accuracy for this new threshold for classification of IO Score positive patients into the IM subtype was 87%. There was no statistical difference between the calculated threshold and the previously established threshold of 0.09 (p = 0.42, t-test of proportions). Based on these data, we continued to implement the pre-defined threshold of 0.09 previously validated in TNBC and NSCLC to analyze the clinical response data from the IMvigor210 clinical trial cohort.

**Fig. 4:**
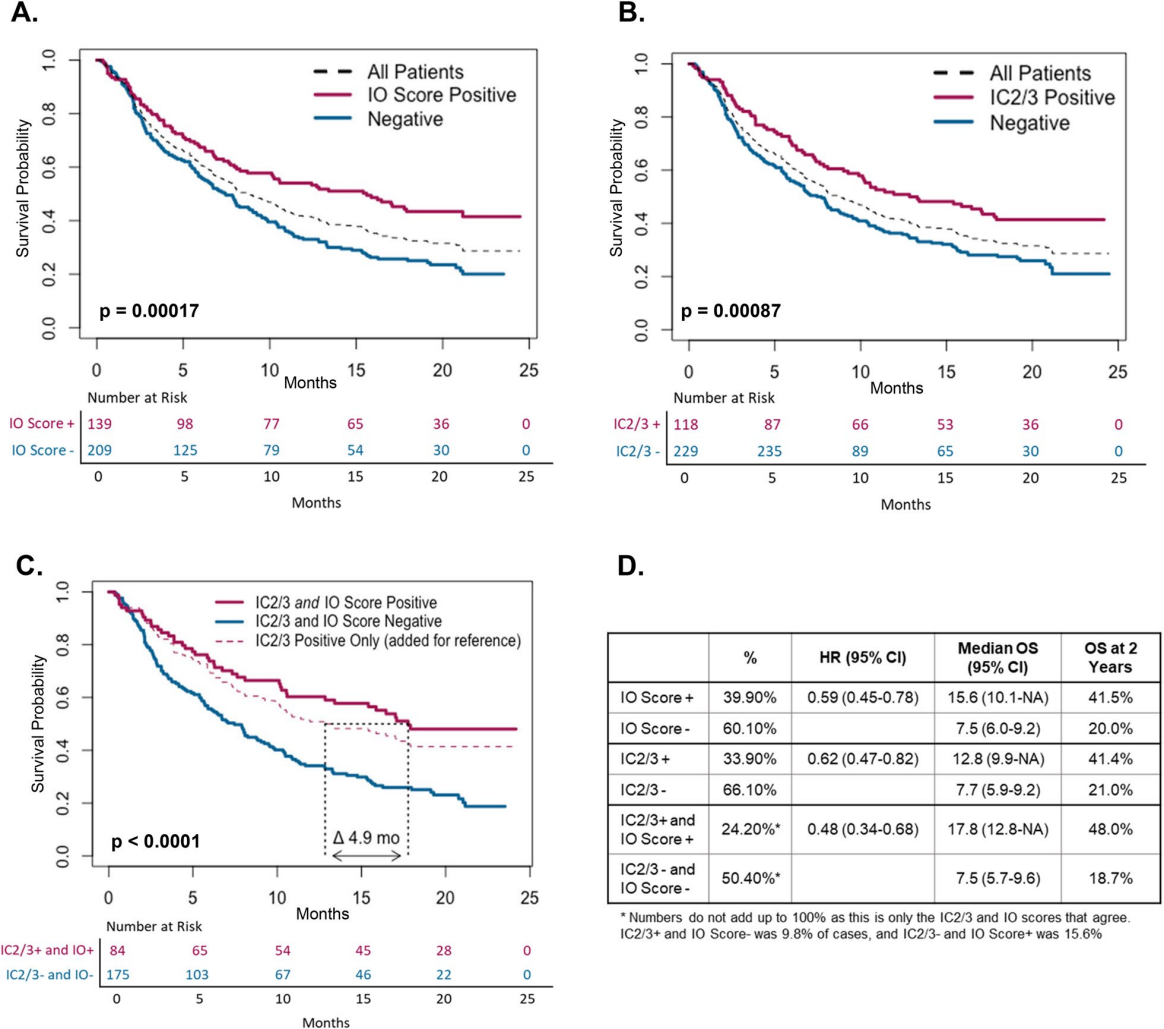
Kaplan Meier Curves for IO Score, PD-L1 IC2/3, and Combined Dual-Positive Cases **A** Kaplan Meier curves for IO Score with HR and OS results; **B** Kaplan Meier curves for IC2/3 with HR and OS results; **C** Kaplan Meier curves for combined analysis of IO Score-positive AND IC2/3-postive cases with HR and OS results. Dual positive patients had a median OS of 17. 8 months compared to 12.8 (difference of 4.9 months) and an HR of 0.48 compared to 0.62. **D** Summary statistics for IO for the three Kaplan–Meier estimates

The IMvigor210 combined platinum resistant and platinum contraindicated patients for a total of 429 subjects of which 348 patients were successfully RNA sequenced [4]. For these 348 patients, the median follow-up time was 20.6 months (95% CI 18.0–21.7) and the median survival was 8.8 months (95% CI 7.4–10.6). OS at the end of study (24.5 months) was 28.6% (95% CI 23.8–34.5%). In total, 40% of the genomic cohort had positive IO Scores. Median OS was 15.6 months (95% CI 10.0-NR) for IO Score positive patients versus 7.5 months (95% CI 6.0–9.2) for IO Score negative patients (Fig. 4A). IC2/3 positive subjects comprised 34% of the cohort. IC2/3 positive patients had a median survival of 12.8 months (95% CI 9.9-NA) versus 7.7 (95% CI 5.9–9.2) for IC2/3 negative subjects (Fig. 4B). The HRs for IO Score and IC2/3 positive subjects were similar as was the percentage of patients alive at end of study: HR for OS at the end of the study for IO Score and IC2/3 were 0.59 (95% CI 0.45–0.78, p < 0.001) and 0.62 (95% CI 0.47–0.82), respectively. At the end of the study, 41.5% (95% CI 33.5–51.4%) of IO positive subjects and 20% (95% CI 14.6–27.5%) of IO Score negative subjects were still alive. Similarly, 41.4% (33.2–51.6%) of IC2/3 positive subjects and 21% (15.4–28.7%) of IC2/3 negative subjects were alive at end of study.

Given the failure of the IMvigor211 to reach the primary endpoint of improved OS in IC2/3 patients, we performed an exploratory analysis to determine if IC2/3 positive patients who were also IO Score positive has better OS (Fig. 4C). These “double positive” patients were 24% of the total cohort, had a median OS of 17.8 months (12.8-NA) with an HR for OS of 0.48 (95% CI 0.34–0.68) and an end of study survival of 48% (95% CI 38.2–60.5%). “Double negative” patients were 50% of the population with median OS of 7.5 month (95% CI 5.7–9.6) and end of study survival of 18.7% (95% CI 12.9–27.2%).

In order to test whether IO Score was quantitatively related to objective response, the continuous IO Score was plotted against the four objective response categories (Additional file 1: Figure S1). The scores were significantly different when comparing the average IO continuous score for patients with a CR to the average of the continuous score patients with a PR, SD, and PD (p < 0.005), when response was compared to non-response (CR/PR versus SD/PD, p < 0.005), or when disease control was compared to progressive disease (CR/PR/SD to PD, p < 0.01). There was also a significant ordinal trend for IO Score across the objective response categories when ordered by decreasing response (p < 0.001).

**Fig. 5:**
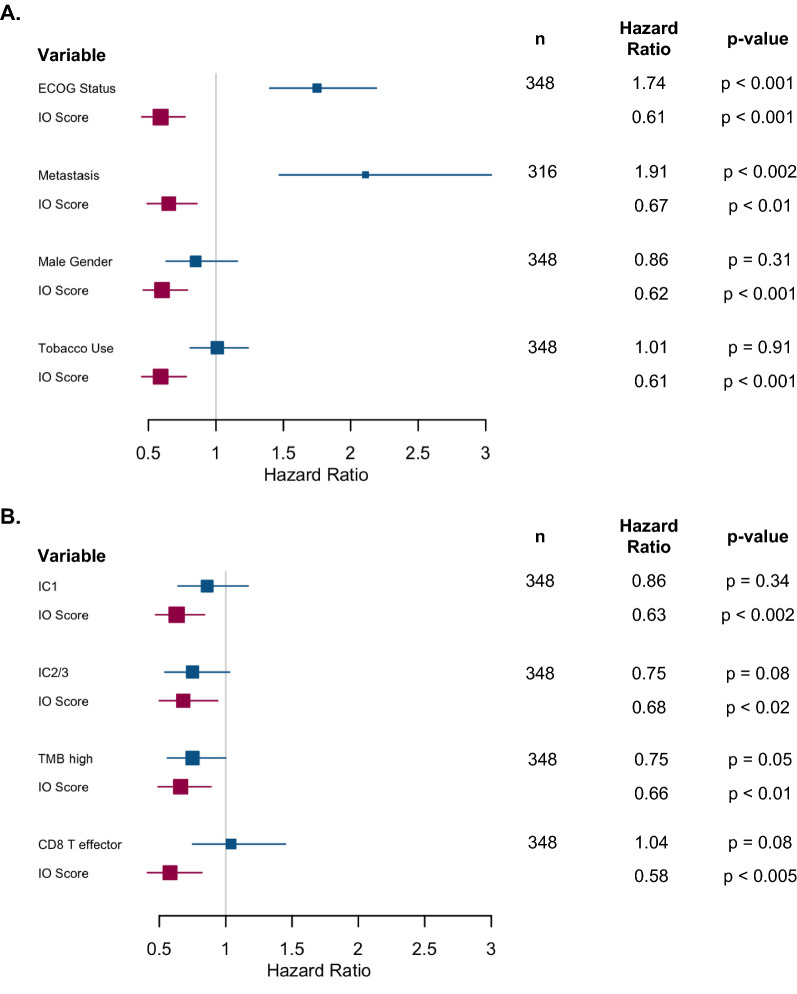
IO Score Independence with Various Clinical Factors and Genomic Biomarkers **A** Series of bivariate Cox proportional hazards with various Clinical Factors. (Note ECOG Status (0, 1, and 2) and Tobacco Use (Never Smokers versus Previous Smokers versus Current Smokers) were treated as increasing risk factors. Metastasis was categorized as lymph node involvement versus either liver or visceral metastasis.); **B** Series of bivariate Cox proportional hazards with various Genomic Biomarkers. IC1 and IC2/3 is PD-L1 staining in immune cells of  > 1% and  > 5% of cells, respectively. TMB-high and TMB-low refer TMB  ≥ 10 mutations and  < 10 mutations per megabase, respectively. The IO Score maintained its significance in every analysis

Mariathasan et al. explored 28 different published clinical factors and biomarker signatures in the IMvigor210 cohort [10], including nineteen genomic signatures. IO Score was tested in a series of bivariate equations with each of these factors and maintained significance with every explored biomarker (Fig. 5A and B and Additional file 2: Figure S2A, Additional file 3: Figure S2B, Additional file 4: Figure S2C) and five of these biomarkers maintained significance with the IO Score. Of the standard clinical factors, ECOG status and regional versus distant metastasis maintained significance with the IO Score. Of the twenty-one genomic signatures, two maintained significance with the IO Score—a previously established, prognostic bladder cancer classifier (Lund classification [37, 38] HR = 1.08 p < 0.025 classification) and a cell cycle regulatory signature (HR = 1.34 p < 0.05). IC2/3 narrowly missed significance for OS in this cohort (HR = 0.53 p = 0.06). TMB was independent of IO Score when using either the previously FDA pan-cancer established threshold of  ≥ 10 mut/MB (TMB-high) (HR = 0.65 p < 0.01) or the study specific top quartile threshold fit to the IMvigor210 dataset (HR = 0.53 p < 0.005) (Additional file 5: Figure S3A). Of note, in the IO Score/TMB-high multivariate analysis, neither HR was significantly different from its univariate HR (univariate HRs 0.59/0.57 for IO Score/TMB-high, respectively; multivariate HRs 0.57/0.65) leading to the exploratory analysis of a decision tree model, where a patient was deemed “positive” if they were IO Score + or TMB-high and “negative” if they were neither (Additional file 5: Figure S3B). The HR for the decision tree model (n = 272) was similar, equaling 0.579 (95% CI 0.429–0.782, p < 0.001) with the key difference being the percentage of patients identified as positive: 61% by the model versus 40% and 41% for the IO Score and TMB-high respectively [Additional file 5: Figure S3C], with 22% of patients being positive for both IO Score and TMB-high.

## Discussion

An ideal biomarker for predicting ICI benefit would perform well across many tumor types and treatment indications, would be independent of clinicopathologic features, and would improve on currently established biomarkers. The IO Score has been previously shown to be associated with improved response to ICI therapy in NSCLC and TNBC [11–15]. Consistent with the hypothesis that the IO Score classifies three components of the TIME that are common to tumors of epithelial origin, IM, MSL (linked with the expression of cancer associated fibroblasts) and M, we tested whether the IO Score would be applicable to classification of bladder cancer.

We first confirmed the TIME classification function, using our previously established threshold for binary classification, to distinguish those strongly expressing the IM signature from those which were more enriched for MSL or M using TCGA-BLCA gene expression data in ICI-naïve patients. We then tested this validated algorithm and threshold for an association with clinical response to ICI therapy in the IMvigor210 bladder cancer clinical trial cohort. The strong association of the IO Score with OS was independent of other explored biomarkers, including both TMB and PD-L1.

The IMvigor210 study resulted in an accelerated, non-biomarker contingent approval for atezolizumab in platinum-refractory patients but was later withdrawn based on a negative confirmatory trial in the IC2/3 biomarker selected patient population (IMvigor211) [9]. This withdrawal highlights the need for careful selection of a biomarker that demonstrates consistent biology when being used for patient selection in a trial. We hypothesize that using a biomarker such as IO Score to enrich for likely responders would minimize the risk of clinical trial failures. Interestingly, one potential use of the IO Score is to combine it with other biomarkers. In the case of IC2/3, the combination of “double positives” increased median survival by 4.9 months, decreased the HR by 0.12, and increased the percentage of patients alive at end of study by 7% over IC2/3 alone. In the case of TMB and IO Score, where the bivariate analysis showed both markers were independent with little change in HR, the analysis was the inverse; a patient was deemed “positive” if he or she were positive for at least one of the biomarkers. This decision tree model led to an increase in the number of patients deemed positive overall (61% versus the 40% and 41% for IO Score and TMB-high, respectively) with little loss in clinical performance. Mariathasan et al. [10] attempted to identify biomarkers from the IMvigor210 trial to enrich for likely responders and yielded several potential candidates. While several of these signatures were significant in univariate models, one striking observation from our study is that of the 21 signatures tested, only two signatures, the Lund signature, and a cell-cycle regulator signature, maintained independence with IO Score (Fig. 5 and Additional file 2: Figure S2).

The demonstrated clinical utility of the IO Score may be due to the unsupervised clustering performed to first identify robust classifiers before proceeding to building the algorithm. This approach to biomarker development is similar to the approach taken by Perou and colleagues who demonstrated the use of hierarchical clustering of gene expression data to identify likely responders to endocrine therapy within hormone positive breast cancer. Their work led to the development of a targeted biomarker panel, PAM50 [39], now an FDA 510 (k) cleared assay to help inform use of adjuvant chemotherapy in addition to endocrine therapy. Partially because PAM50 was built on a robust classifier based on shared components between cancer types, it was confirmed and further defined as an informative biomarker in hormone-sensitive prostate cancer [40, 41].

Given our prior approach and published data in TNBC and NSCLC, we believe that by assessing the IO Score as a classifier of the TIME in ICI naïve patients, we can better inform clinical decision making for mUC patients due to the IO Score’s strong association with response to ICI therapy in multiple tumor types. Future studies in both bladder cancers and additional tumor types using randomized clinical trial samples are warranted.

## Supplementary Information

**Additional file 1: Figure S1.** Average score for the continuous IO Score by response The IO Score was significant by trend (ordinal logistic regression), complete response, objective response, and disease control rate (horizontal lines from top to bottom). CR = complete response, PR = partial response, SD = stable disease, and PD = progressive disease. Objective Response is CR or PR versus SD or PD and Disease Control is CR, PR, or SD versus PD. **p≤0.01, ***p≤0.001.

**Additional file 2: Figure S2A.**. IO Score Independence with Additional Clinical Factors and Genomic Biomarkers Demonstrating IO Score independence with various genomic signatures in a series of bivariate Cox Proportional Hazards. In all cases the median of the signature was used as a threshold for positive or negative. A more complete description of each of these signatures can be found in the work of Mariathasan and colleagues [10].

**Additional file 3. Figure S2B.** IO Score Independence with Additional Clinical Factors and Genomic Biomarkers Demonstrating IO Score independence with various genomic signatures in a series of bivariate Cox Proportional Hazards. In all cases the median of the signature was used as a threshold for positive or negative. A more complete description of each of these signatures can be found in the work of Mariathasan and colleagues [10].

**Additional file 4. Figure S2C.** IO Score Independence with Additional Clinical Factors and Genomic Biomarkers Demonstrating IO Score independence with various genomic signatures in a series of bivariate Cox Proportional Hazards. In all cases the median of the signature was used as a threshold for positive or negative. A more complete description of each of these signatures can be found in the work of Mariathasan and colleagues [10].

**Additional file 5: Figure S3A. **Identifying Additional Eligible Patients by Combining IO Score and TMB **(A)** Kaplan Meier curves showing individual OS results for TMB-high. **(B)** Kaplan Meier curves showing the combined results considering patients that were either IO Score+ or TMB -high versus those that were negative for both. **(C)** Percentage of patients positive for TMB-high/IO Score, and a combined population of either TMB-high or IO Score+. **(D)** Median and 2-year OS rates for each individual marker and combined population of either IO Score+ or TMB-high.

## Data Availability

The gene expression from TCGA (HT-Seq) is publicly available at the Genomic Data Commons Data Portal. The data used here was downloaded on April 20, 2020 (TCGA-BRCA), May 2, 2020 (TCGA-LUSQ and TCGA-LUAD) and December 20, 2020 (TCGA-BLCA), all raw sequencing data required for the IMVigor210 RNA-seq analyses were previously deposited by Mariathasan et al. to the European Genome-Phenome Archive under accession number EGAS00001002556. The source code and processed data used for all analyses presented here have been made available in *IMvigor210CoreBiologies* (see Methods).

## Abbreviations

CI: Confidence interval
CR: Complete response
EMT: Epithelial-to-mesenchymal transition
FDA: Food and drug administration
HGNC: HUGO Gene Nomenclature Committee
HR: Hazard ratio
HUGO: Human Genome Organisation
IC1: 1% or higher of immune cells are positive PD-L1 IHC staining
IC2/3: 5% or higher of immune cells are positive PD-L1 IHC staining
ICI: Immune checkpoint inhibitor
IHC: Immunohistochemistry
IM: Immunomodulatory
IO: Immuno-oncology
M: Mesenchymal
MSL: Mesenchymal stem-like
mUC: Metastatic urothelial cancer
NSCLC: Non-small cell lung cancer
OS: Overall survival
PD: Progressive disease
PD-1: Program cell death-1
PD-L1: Program cell death ligand-1
PR: Partial response
SD: Stable disease
TCGA: The Cancer Genome Atlas
TCGA-BLCA: TCGA bladder carcinoma gene expression dataset
TCGA-BRCA: TCGA breast carcinoma gene expression dataset
TCGA-LUAD: TCGA lung adenocarcinoma gene expression dataset
TCGA-LUSQ: TCGA lung squamous cell carcinoma gene expression dataset
TIME: Tumor immune microenvironment
TMB: Tumor mutation burden
TMB-high: Tumor mutation burden greater than or equal to 10 mutations per megabase
TMB-low: Tumor mutation burden less than 9 mutations per megabase
TNBC: Triple negative breast cancer
