# Supplementary figures and images for: Translation of the 27-gene immuno-oncology test (IO score) to predict outcomes in immune checkpoint inhibitor treated metastatic urothelial cancer patients
Source: J Transl Med. 2022 Aug 16;20:370. doi: 10.1186/s12967-022-03563-9 (PMC9382843; doi:10.1186/s12967-022-03563-9)

**A.**

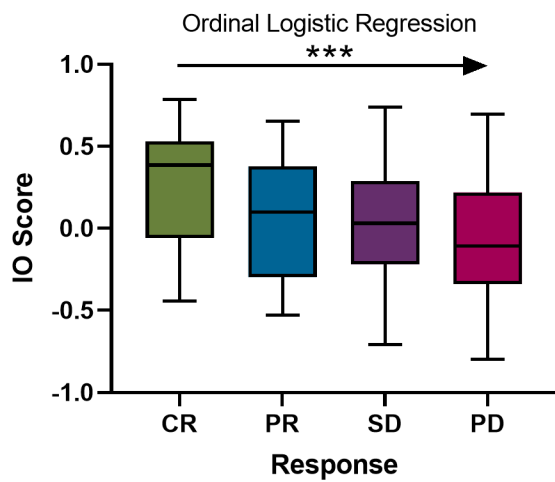

**B.**

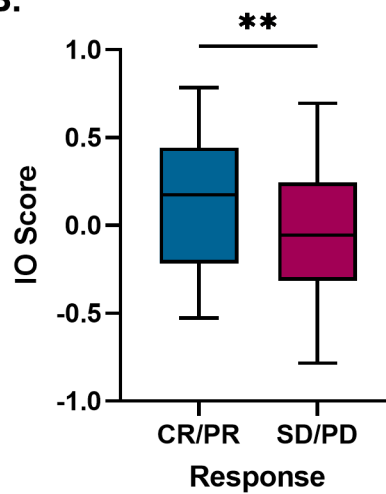

**C.**

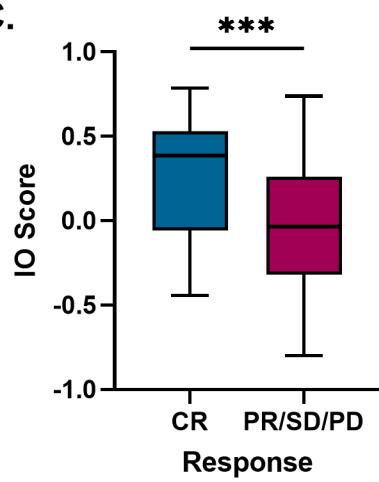

**D.**

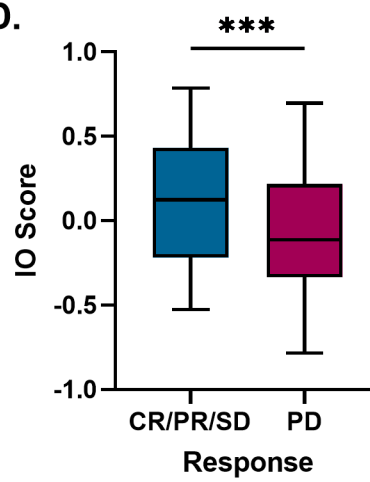

Supplement: Supplementary file 1 — Additional file 1: Figure S1. Average score for the continuous IO Score by response The IO Score was significant by trend (ordinal logistic regression), complete response, objective response, and disease control rate (horizontal lines from top to bottom). CR = complete response, PR = partial response, SD = stable disease, and PD = progressive disease. Objective Response is CR or PR versus SD or PD and Disease Control is CR, PR, or SD versus PD. **p≤0.01, ***p≤0.001. [file 12967_2022_3563_MOESM1_ESM.pdf]
