# Supplementary material for: Translation of the 27-gene immuno-oncology test (IO score) to predict outcomes in immune checkpoint inhibitor treated metastatic urothelial cancer patients
Source: J Transl Med. 2022 Aug 16;20:370. doi: 10.1186/s12967-022-03563-9 (PMC9382843; doi:10.1186/s12967-022-03563-9)

A.

Variable	n	Hazard Ratio	p-Value
Lund Index	348	1.08	$p < 0.05$
IO Score		0.56	$p < 0.001$
DNA damage repair	348	0.88	$p = 0.34$
IO Score		0.63	$p < 0.002$
Antigen processing machinery	348	1.32	$p = 0.08$
IO Score		0.51	$p < 0.001$
Immune checkpoint	348	1.03	$p = 0.86$
IO Score		0.60	$p < 0.002$
Cell cycle regulators	348	1.34	$p < 0.05$
IO Score		0.66	$p < 0.005$
Fanconi anemia	348	0.89	$p = 0.38$
IO Score		0.63	$p < 0.001$
Pan-F-TBRS	348	1.13	$p = 0.37$
IO Score		0.62	$p < 0.001$

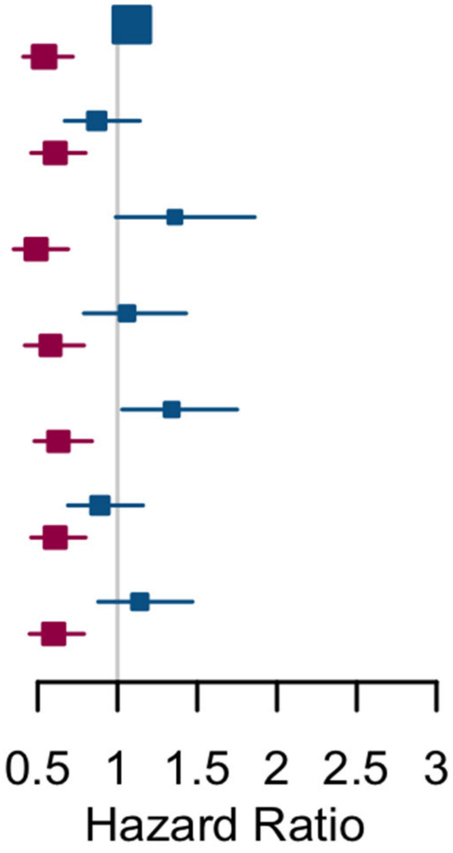

Supplement: Supplementary file 2 — Additional file 2: Figure S2A.. IO Score Independence with Additional Clinical Factors and Genomic Biomarkers Demonstrating IO Score independence with various genomic signatures in a series of bivariate Cox Proportional Hazards. In all cases the median of the signature was used as a threshold for positive or negative. A more complete description of each of these signatures can be found in the work of Mariathasan and colleagues [10]. [file 12967_2022_3563_MOESM2_ESM.pdf]
