# Supplementary material for: Translation of the 27-gene immuno-oncology test (IO score) to predict outcomes in immune checkpoint inhibitor treated metastatic urothelial cancer patients
Source: J Transl Med. 2022 Aug 16;20:370. doi: 10.1186/s12967-022-03563-9 (PMC9382843; doi:10.1186/s12967-022-03563-9)

B.

Variable	n	Hazard Ratio	p-Value
TCGA Signature	348	0.87	p =0.32
IO Score		0.60	p < 0.001
Kegg Histone Signature	348	0.89	p = 0.39
IO Score		0.63	p < 0.001
EMT 1	348	1.18	p = 0.22
IO Score		0.61	p < 0.001
EMT 2	348	1.27	p = 0.07
IO Score		0.62	p < 0.001
EMT 3	348	1.18	p = 0.22
IO Score		0.61	p < 0.001
WNT target	348	1.10	p = 0.48
IO Score		0.64	p < 0.005
FGFR3-related genes	348	1.11	p = 0.42
IO Score		0.62	p < 0.001

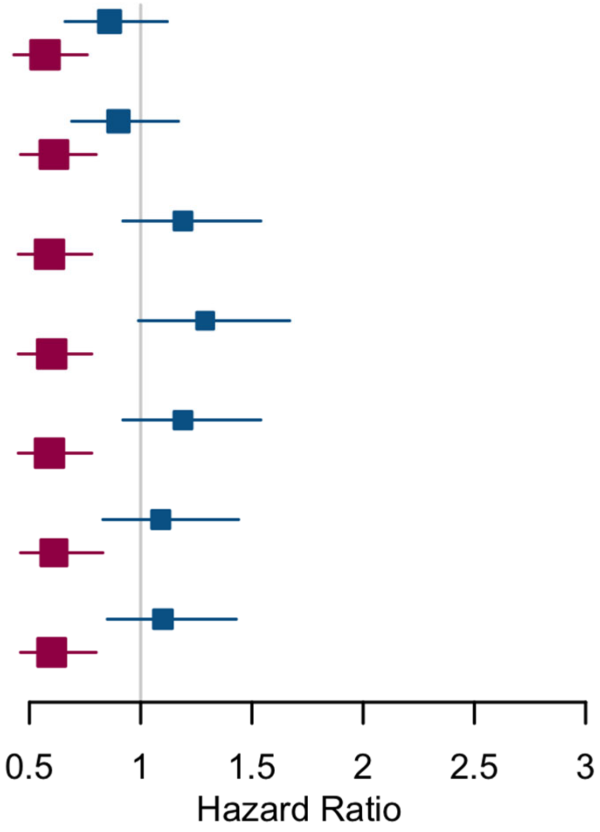

Supplement: Supplementary file 3 — Additional file 3. Figure S2B. IO Score Independence with Additional Clinical Factors and Genomic Biomarkers Demonstrating IO Score independence with various genomic signatures in a series of bivariate Cox Proportional Hazards. In all cases the median of the signature was used as a threshold for positive or negative. A more complete description of each of these signatures can be found in the work of Mariathasan and colleagues [10]. [file 12967_2022_3563_MOESM3_ESM.pdf]
