# Supplementary material for: Translation of the 27-gene immuno-oncology test (IO score) to predict outcomes in immune checkpoint inhibitor treated metastatic urothelial cancer patients
Source: J Transl Med. 2022 Aug 16;20:370. doi: 10.1186/s12967-022-03563-9 (PMC9382843; doi:10.1186/s12967-022-03563-9)

C.

Variable	n	Hazard Ratio	p-Value
Cell cycle	348	0.94	p = 0.63
IO Score		0.62	p < 0.001
Mismatch repair	348	0.99	p = 0.95
IO Score		0.61	p < 0.001
Homologous recombination	348	0.86	p = 0.25
IO Score		0.63	p < 0.002
Nucleotide excision repair	348	1.03	p = 0.81
IO Score		0.61	p < 0.001
DNA replication	348	0.97	p = 0.83
IO Score		0.62	p < 0.001
Base excision repair	348	1.07	p = 0.59
IO Score		0.60	p < 0.001

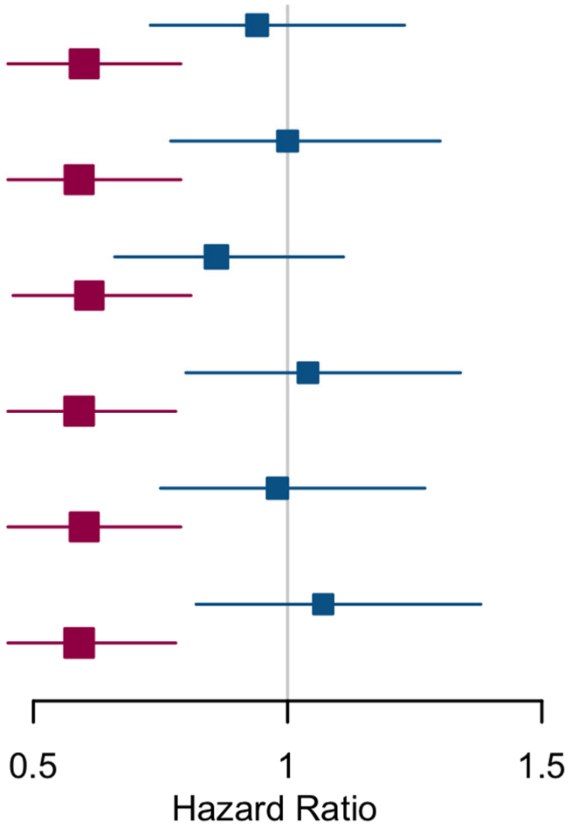

Supplement: Supplementary file 4 — Additional file 4. Figure S2C. IO Score Independence with Additional Clinical Factors and Genomic Biomarkers Demonstrating IO Score independence with various genomic signatures in a series of bivariate Cox Proportional Hazards. In all cases the median of the signature was used as a threshold for positive or negative. A more complete description of each of these signatures can be found in the work of Mariathasan and colleagues [10]. [file 12967_2022_3563_MOESM4_ESM.pdf]
