# Supplementary material for: Translation of the 27-gene immuno-oncology test (IO score) to predict outcomes in immune checkpoint inhibitor treated metastatic urothelial cancer patients
Source: J Transl Med. 2022 Aug 16;20:370. doi: 10.1186/s12967-022-03563-9 (PMC9382843; doi:10.1186/s12967-022-03563-9)
Supplement: Supplementary file 5 — Additional file 5: Figure S3A. Identifying Additional Eligible Patients by Combining IO Score and TMB (A) Kaplan Meier curves showing individual OS results for TMB-high. (B) Kaplan Meier curves showing the combined results considering patients that were either IO Score+ or TMB -high versus those that were negative for both. (C) Percentage of patients positive for TMB-high/IO Score, and a combined population of either TMB-high or IO Score+. (D) Median and 2-year OS rates for each individual marker and combined population of either IO Score+ or TMB-high. [file 12967_2022_3563_MOESM5_ESM.pdf]

A.

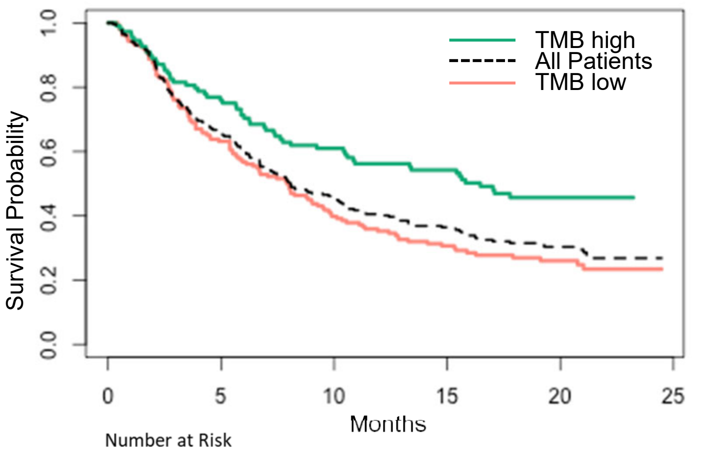

Number at Risk

	0	5	10	15	20	25
TMB high	112	82	64	55	31	0
TMB low	160	98	61	43	25	0

Months

B.

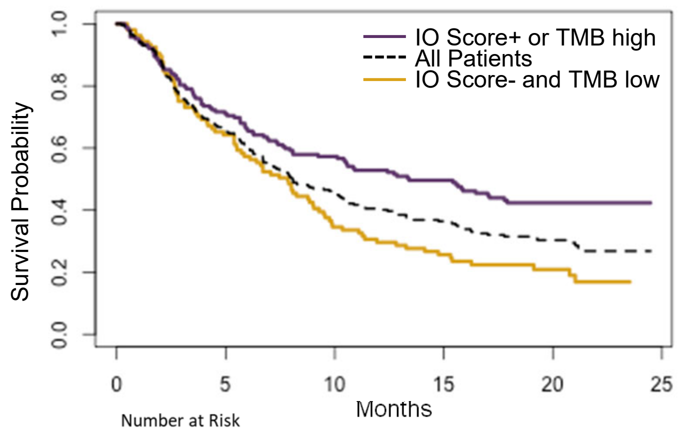

Number at Risk

	0	5	10	15	20	25
TMB high or IO Score +	164	115	90	74	43	0
IO Score - and TMB low	108	65	35	24	13	0

Months

C.

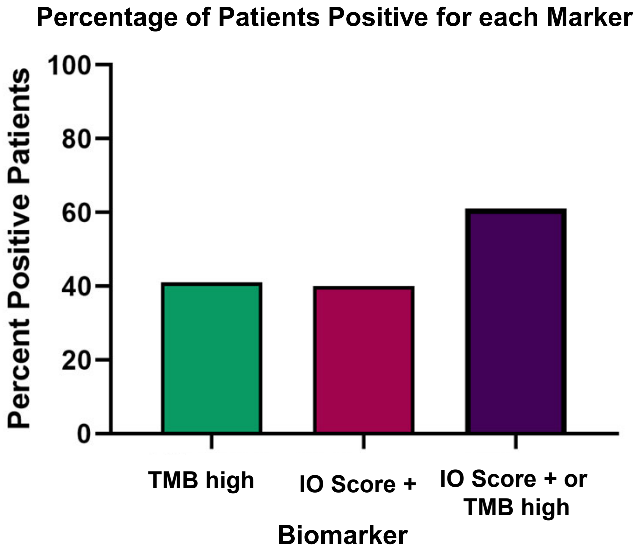

D.

Median and 2-year OS for each Marker and the Combined Population

	Median OS (mos)	2 Year OS
IO Score + or TMB high	13.4	42.3%
IO Score +	15.6	41.5%
TMB high	16.5	45.7%
